# Fatty acids stimulate insulin secretion from human pancreatic islets at fasting glucose concentrations via mitochondria-dependent and -independent mechanisms

**DOI:** 10.1186/s12986-016-0119-5

**Published:** 2016-08-30

**Authors:** Jing Cen, Ernest Sargsyan, Peter Bergsten

**Affiliations:** Department of Medical Cell Biology, Uppsala University, Box 571, Uppsala, Sweden

**Keywords:** Insulin secretion, Human pancreatic islets, Saturated fatty acids, Monounsaturated fatty acids, Mitochondrial respiration

## Abstract

**Background:**

Free fatty acids (FFAs) acutely stimulate insulin secretion from pancreatic islets. Conflicting results have been presented regarding this effect at non-stimulatory glucose concentration, however. The aim of our study was to investigate how long-chain FFAs affect insulin secretion from isolated human pancreatic islets in the presence of physiologically fasting glucose concentrations and to explore the contribution of mitochondria to the effects on secretion.

**Methods:**

Insulin secretion from human pancreatic islets was measured from short-term static incubation or perfusion system at fasting glucose concentration (5.5 mM) with or without 4 different FFAs (palmitate, palmitoleate, stearate, and oleate). The contribution of mitochondrial metabolism to the effects of fatty acid-stimulated insulin secretion was explored.

**Results:**

The average increase in insulin secretion, measured from statically incubated and dynamically perifused human islets, was about 2-fold for saturated free fatty acids (SFAs) (palmitate and stearate) and 3-fold for mono-unsaturated free fatty acids (MUFAs) (palmitoleate and oleate) compared with 5.5 mmol/l glucose alone. Accordingly, MUFAs induced 50 % and SFAs 20 % higher levels of oxygen consumption compared with islets exposed to 5.5 mmol/l glucose alone. The effect was due to increased glycolysis. When glucose was omitted from the medium, addition of the FFAs did not affect oxygen consumption. However, the FFAs still stimulated insulin secretion from the islets although secretion was more than halved. The mitochondria-independent action was via fatty acid metabolism and FFAR1/GPR40 signaling.

**Conclusions:**

The findings suggest that long-chain FFAs acutely induce insulin secretion from human islets at physiologically fasting glucose concentrations, with MUFAs being more potent than SFAs, and that this effect is associated with increased glycolytic flux and mitochondrial respiration.

## Background

Short-term exposure of beta-cells to free fatty acids (FFAs) potentiates glucose-stimulated insulin secretion [1, 2]. Such potency has been reported to increase with chain length and degree of saturation of the FFAs [2, 3]. The effect of FFAs on insulin secretion has been related to FFA metabolism [4–6] and to signaling via Gq protein-coupled receptor, FFAR1/GPR40 [7, 8]. Our recent studies demonstrated that in the presence of elevated glucose concentrations rise in insulin secretion is associated with increased mitochondrial function, which in turn is mainly attributed to enhanced glucose oxidation [9].

FFAs of different chain length and degrees of saturation are normal components in the circulation [10, 11] and their levels are raised during the fasting state [12]. At low glucose concentrations, FFAs are main substrates for energy production in islets [13, 14]. Whether FFAs affect insulin secretion at fasting glucose levels is not clear. Whereas some groups reported that there is no or little effect of FFAs on insulin secretion [3, 15, 16], others showed that FFAs stimulate insulin secretion at low glucose concentrations [17, 18].

In the present study we have addressed how long-chain saturated free fatty acids (SFAs) palmitate and stearate and their corresponding mono-unsaturated free fatty acids (MUFAs) palmitoleate and oleate affect insulin secretion from isolated human pancreatic islets at fasting glucose concentrations. These FFAs are four of the most prevalent fatty acids in the circulation [11]. We also explored the role of mitochondrial activity in the action. We found that FFAs acutely increased insulin secretion at fasting glucose concentrations with MUFAs being more potent than SFAs, and that the enhanced insulin secretion was partly associated with the rise in glucose flux and mitochondrial respiration.

## Methods

### Human islet culture

Human islets were obtained from the Nordic Network for Clinical Islet Transplantation (Uppsala University Hospital, Uppsala, Sweden). In total, human islets from 26 brain-dead non-diabetic donors were used in this study (age: 60.1 ± 2.0 years, male/female: 14/12; BMI: 27.4 ± 0.9 kg/m^2^, HbA1c: 5.5 ± 0.1 %). Human islets were cultured in CMRL 1066 medium (Invitrogen, Paisley, UK) containing 5.5 mmol/l glucose (Sigma, St. Louis, MO) and supplemented with 10 % fetal bovine serum (Invitrogen), 1 % glutamine (Invitrogen), 100 units/ml penicillin (Invitrogen) and 100 μg/ml streptomycin (Invitrogen) at 37 °C in humidified air containing 5 % CO_2_. Islets were used within 10 days after isolation.

### Fatty acid preparation

Fatty acids were prepared as previously described [19]. Briefly, 100 mmol/l stock solutions containing palmitate, stearate, or oleate (all from Sigma Aldrich, St. Louis, MO, USA) were prepared by dissolving fatty acids in 50 % ethanol. Stock solution of palmitoleate (Sigma Aldrich) was prepared in 100 % ethanol to a concentration of 200 mmol/l. Stock solutions were then diluted in incubation medium containing 5 mg/ml of fatty acid-free BSA (Boehringer Mannheim GmbH, Mannheim, Germany) to a final concentration of 0.5 mmol/l. Fatty acids were allowed to complex with BSA at 37 °C for at least 30 min.

### Human islet perifusion and static incubation

Human islets were perifused as described previously [20]. Briefly, 25–30 human islets were hand-picked and placed into a perifusion chamber. Islets were perifused for 60 min at 37 °C in KRBH buffer consisting of 130 mmol/l NaCl, 4.8 mmol/l KCl, 1.2 mmol/l MgSO_4_, 1.2 mmol/l KH_2_PO_4_, 1.2 mmol/l CaCl_2_, 5.0 mmol/l NaHCO_3_ and 5.0 mmol/l HEPES, titrated to pH 7.4 with NaOH and supplemented with 5 mg/ml fatty acid-free BSA and 5.5 mmol/l glucose or without glucose. After this initial perifusion period, samples were collected every 5 min for 20 min at the same concentration of glucose. This was followed by another 20-min perifusion with the same buffer containing 5 mg/ml fatty acid-free BSA and 0.5 mmol/l palmitate (16:0), palmitoleate (16:1), stearate (18:0), or oleate (18:1). In addition, 10 μM triacsin C (Sigma Aldrich) or 10 μM DC260126 (Tocris Bioscience, Bristol, UK) were added during the perifusion to inhibit long-chain fatty acyl CoA synthetase or FFAR1/GPR40 signaling, respectively. Perifusates were collected at 2, 4, 6, 8, 10, 15, and 20 min. The perifusion rate was 170 μl/min and collected perifusates were used to measure the amounts of secreted insulin. Insulin released during the first 6 min after the introduction of fatty acids was referred to as the first phase and that during the subsequent 14 min was regarded as the second phase.

Human islets were hand-picked in batches of 50 and statically incubated for 60 min in 0.5 ml KRBH buffer, identical to the one used for the perifusion, supplemented with 0.5 mmol/l of the different fatty acids and 5.5 mmol/l glucose or without glucose for 20 min.

The amount of insulin secreted was measured by enzyme-linked immunosorbent assay as described previously [21]. For each perifusion, total fatty acid-induced insulin secretion was normalized to insulin secretion in the absence of the fatty acids. For static incubation total insulin secretion during 20 min was normalized to total protein content and insulin secretion was expressed as fold change between insulin secretion from fatty acid-treated and untreated islets for each donor.

### Oxygen consumption and extracellular acidification rates

The oxygen consumption rate (OCR) and extracellular acidification rate (ECAR) of isolated human pancreatic islets were determined by Extracellular Flux Analyzer XF^e^96 (Seahorse Bioscience, MA, USA) as previously reported [22]. Ten hand-picked human islets were placed into the wells of the XF^e^96 cell culture microplate pre-coated with poly-D-lysine. Islets were pre-incubated with assay medium (Seahorse Bioscience) composed of 143 mmol/l NaCl, 5.4 mmol/l KCl, 0.91 mmol/l NaH_2_PO_4_, 0.8 mmol/l MgSO_4_, 1.8 mmol/l CaCl_2_, 2 mmol/l Glutamax, 3 mg/l Phenol Red, and supplemented with 5 mg/ml fatty acid-free BSA and 0.5 mmol/l palmitate (16:0), palmitoleate (16:1), stearate (18:0), or oleate (18:1) in the presence or absence of 5.5 mmol/l glucose (pH adjusted to 7.4) for 1 h at 37 °C before the microplate was inserted into the Analyzer. For each donor, 6–8 replicates of each treatment condition were used. OCR and ECAR were then measured in parallel for 40 min followed by the injection of 5 μmol/l rotenone and 5 μmol/l antimycin A to inhibit mitochondrial respiration. The remaining OCR was considered as non-mitochondrial respiration. To calculate the mitochondrial respiration, non-mitochondrial OCR was subtracted from the total OCR. Data were normalized to total islet area calculated by the Image J software (National Institutes of Health, USA) from pictures (40×) taken with camera (Olympus) mounted onto an inverted Olympus CKX41 microscope. Calculated basal OCR and ECAR were expressed as fold changes from fatty acid-treated islets compared with that from untreated islets for each experiment.

### Protein content

Human islets were washed with phosphate buffer saline (PBS) (Sigma) and lysed in PBS containing 1 % Triton-X 100 (Sigma). Total protein content in the lysates was determined by DC protein assay according to the manufacturer’s instructions (Bio-Rad Laboratories, USA).

### Data analysis

Results were presented as means ± SEM. Statistical analysis was performed using GraphPad Prism Version 6.0b (GraphPad software, CA, USA). Statistical significance among several groups was analyzed by using one-way ANOVA followed by Holm-Sidak multiple comparison test. *P* < 0.05 was considered statistically significant.

## Results

### Fatty acids acutely enhance insulin secretion, OCR and ECAR from human islets at fasting glucose concentrations with MUFAs being more potent than SFAs

**Fig. 1:**
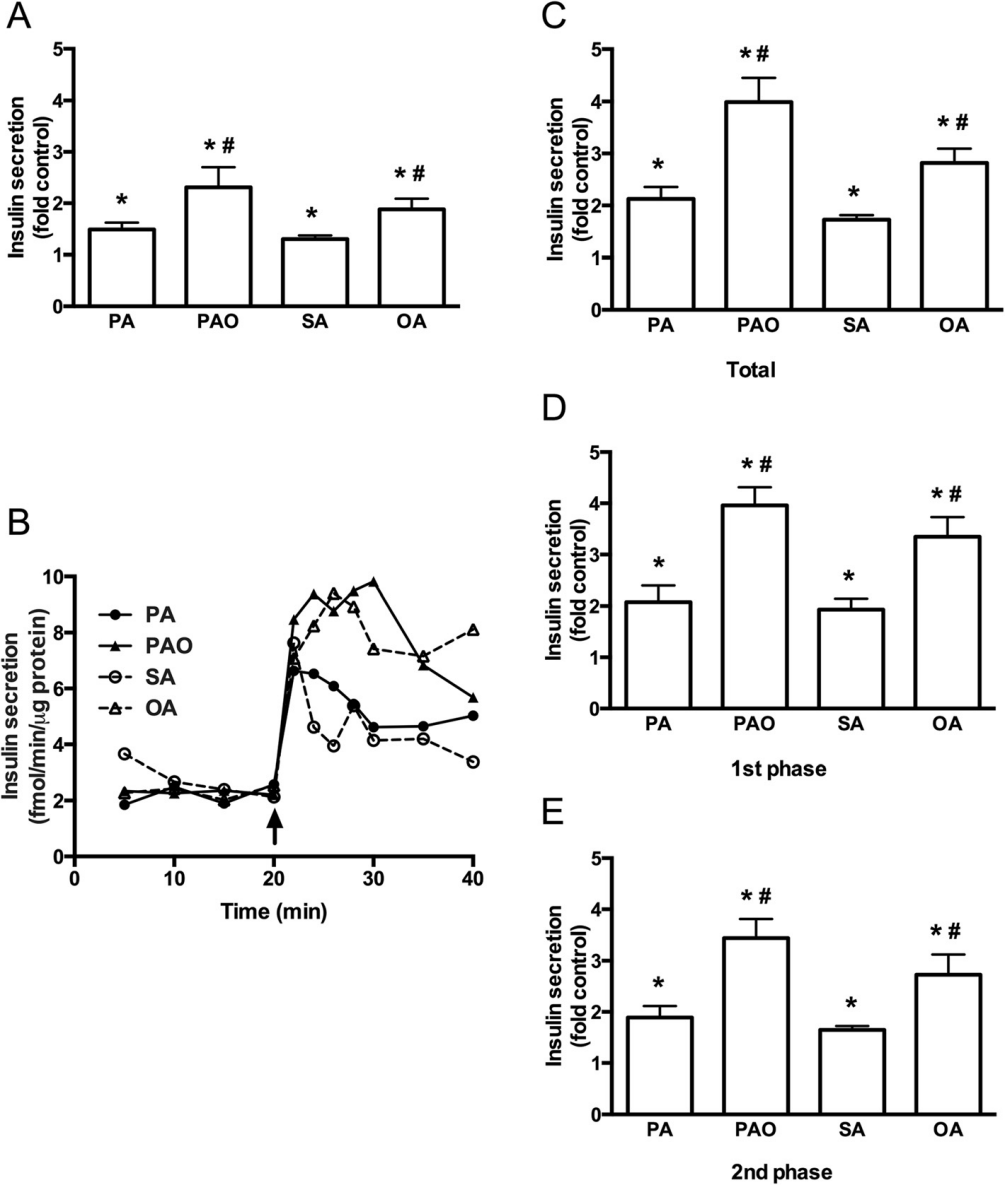
Acute effects of free fatty acids on insulin secretion at 5.5 mmol/l glucose. Human islets were incubated at 5.5 mmol/l glucose in the presence or absence of 0.5 mM palmitate (PA), palmitoleate (PAO), stearate (SA), or oleate (OA) for 20 min. **a** Insulin secretion from statically incubated human islets was expressed as fold secretion at control. **b** Representative graph of dynamic insulin secretion from perifused islets is shown. Fatty acids were added as indicated. **c**-**e** Total, first-phase, and second-phase of fatty acid-stimulated insulin secretion during perifusion was expressed as fold control. Results are means ± SEM of 8 donors. *P < 0.05 vs Control, #*P* < 0.05 PA vs PAO or SA vs OA

Insulin secretion rate from statically incubated human islets in the presence of 5.5 mmol/l glucose was 1.48 ± 0.24 fmol/min/μg protein. When fatty acids were added to the incubation containing 5.5 mmol/l glucose, insulin secretion was significantly raised (Fig. 1a). The rise was around 1.5-fold in the presence SFA palmitate (16:0) or stearate (18:0), and significantly higher reaching 2 to 2.5-fold when exposed to MUFA palmitoleate (16:1) or oleate (18:1). Chain length played no significant role in the effects of fatty acids on insulin secretion. The observations that fatty acids stimulated insulin secretion from human islets in the presence of fasting glucose concentration and that MUFAs were more potent than SFAs made us study the secretory response dynamically by perifusing human islets. Perifused human islets secreted insulin at 2.09 ± 0.12 fmol/min/μg protein in the presence of 5.5 mmol/l glucose (Fig. 1b). When SFA palmitate or stearate was acutely added to the perifusion medium insulin release was enhanced equally by approximately 2-fold by either of the SFAs (Fig. 1b and c). MUFAs were significantly more potent with palmitoleate causing 4-fold and oleate 3-fold rise in insulin secretion (Fig. 1b and c). The rises in insulin secretion caused by SFAs and MUFAs (Fig. 1c) were accounted for by rises in both first (Fig. 1d) and second (Fig. 1e) phases of insulin secretion.

**Fig. 2:**
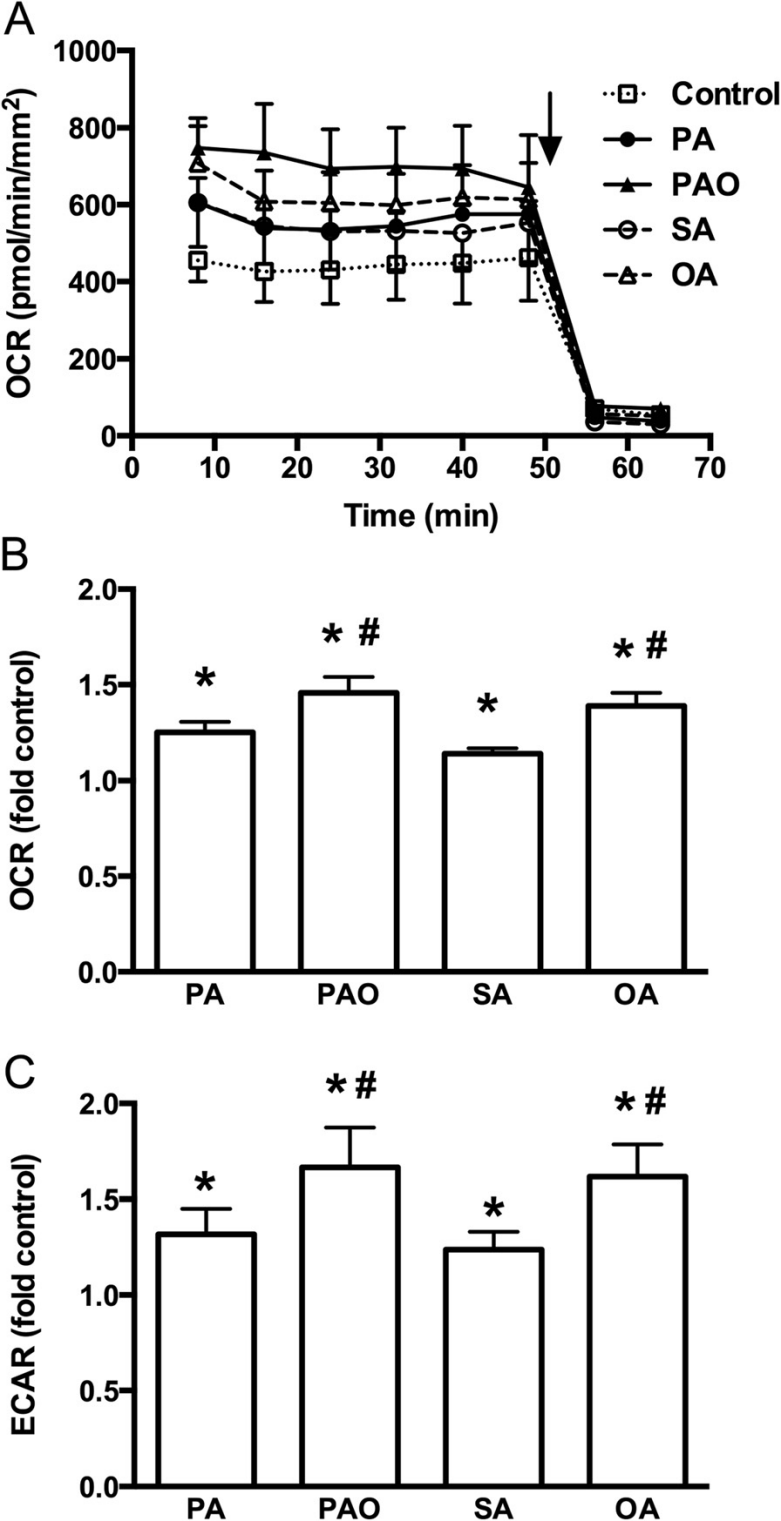
Effects of free fatty acids on metabolism at 5.5 mmol/l glucose. Human islets were incubated at 5.5 mmol/l glucose in the presence or absence of palmitate (PA), palmitoleate (PAO), stearate (SA), or oleate (OA) for 40 min. **a** Representative graph of oxygen consumption rate (OCR) is shown. Rotenone and antimycin A were added as indicated by arrow. **b** and **c** OCR and extracellular acidification rate (ECAR) during 40 min were expressed as fold control. Results are means ± SEM of 4 donors. *P < 0.05 vs Control, #*P* < 0.05 PA vs PAO or SA vs OA

To examine the contribution of mitochondrial metabolism to the effects of SFAs and MUFAs on insulin secretion, mitochondrial OCR during the fatty acid treatment was determined. In the presence of 5.5 mmol/l glucose OCR in human islets was 502 ± 24 pmol/min/mm^2^. The fatty acids significantly elevated OCR with MUFAs being more potent than SFAs (Fig. 2a and b), which was in line with the insulin secretion results (Fig. 1). Whereas SFAs caused 1.2-fold rise in OCR, MUFAs had significantly higher 1.4-fold rise in OCR in human islets.

The contribution of glycolytic flux to the observed changes induced by SFAs and MUFAs on OCR was next examined by measuring ECAR. In the presence of 5.5 mmol/l glucose ECAR in human islets was 99 ± 13 mpH/min/mm^2^. When SFA palmitate or stearate was added, ECAR significantly increased 1.3-fold (Fig. 2c). Further significant rise in ECAR to 1.6-fold was observed, when islets were exposed to MUFA palmitoleate or oleate.

The results suggest that FFAs trigger insulin secretion and enhance mitochondrial respiration in beta-cells with MUFAs more potently than SFAs, and that enhanced glycolysis contributes to these events.

### Fatty acids acutely enhance insulin secretion but not OCR and ECAR from human islets in the absence of glucose with MUFAs being more potent than SFAs

**Fig. 3:**
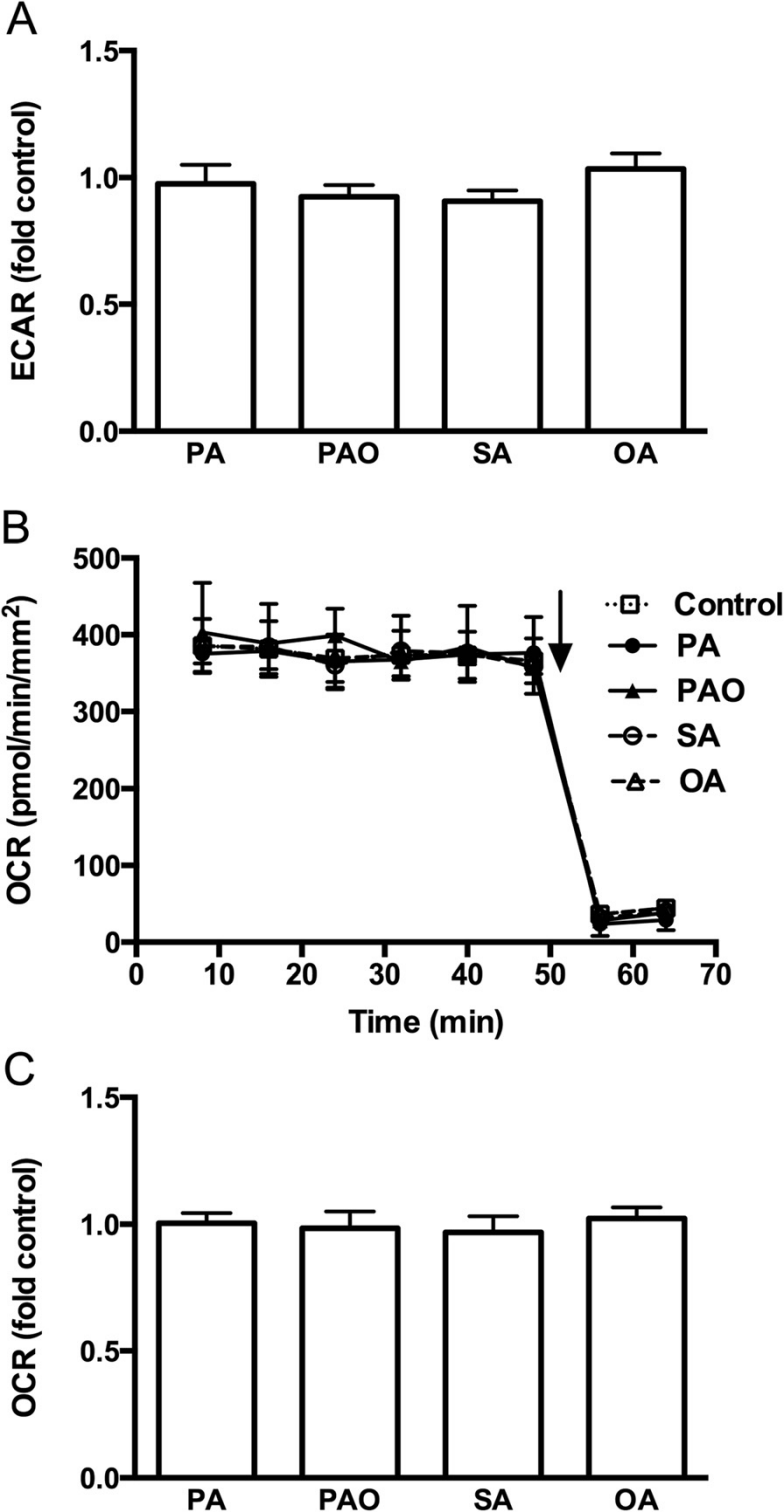
Effects of free fatty acids on metabolism in the absence of glucose. Human islets were incubated with or without palmitate (PA), palmitoleate (PAO), stearate (SA), or oleate (OA) for 40 min in the absence of glucose. **a** Extracellular acidification rate (ECAR) was expressed as fold control. **b** Representative graph of oxygen consumption rate (OCR) is shown. Rotenone and antimycin A were added as indicated by arrow. **c** OCR was expressed as fold control. Results are means ± SEM of 4 donors

To further address the contribution of glycolysis in FFA-induced insulin secretion and rise in OCR, human islets were exposed to the SFA (palmitate or stearate) or MUFA (palmitoleate or oleate) in the absence of glucose. In the absence of glucose and prior to introducing the FFAs, ECAR in the islets was 61 ± 3 mpH/min/mm^2^, which was 60 % of that observed in islets incubated in the presence of 5.5 mmol/l glucose. As expected ECAR was not affected when SFA (palmitate or stearate) or MUFA (palmitoleate or oleate) was added to islets incubated in the absence of glucose (Fig. 3a). When incubating islets in the absence of glucose, OCR was reduced to 440 ± 25 pmol/min/mm^2^, representing 88 % of OCR levels observed in islets incubated in the presence of 5.5 mmol/l glucose. Neither inclusion of the SFAs nor MUFAs caused any change in mitochondrial respiration in these islets maintained in the absence of glucose (Fig. 3b and c). These results confirm the role of glucose in FFA-induced elevation of mitochondrial metabolism.

**Fig. 4:**
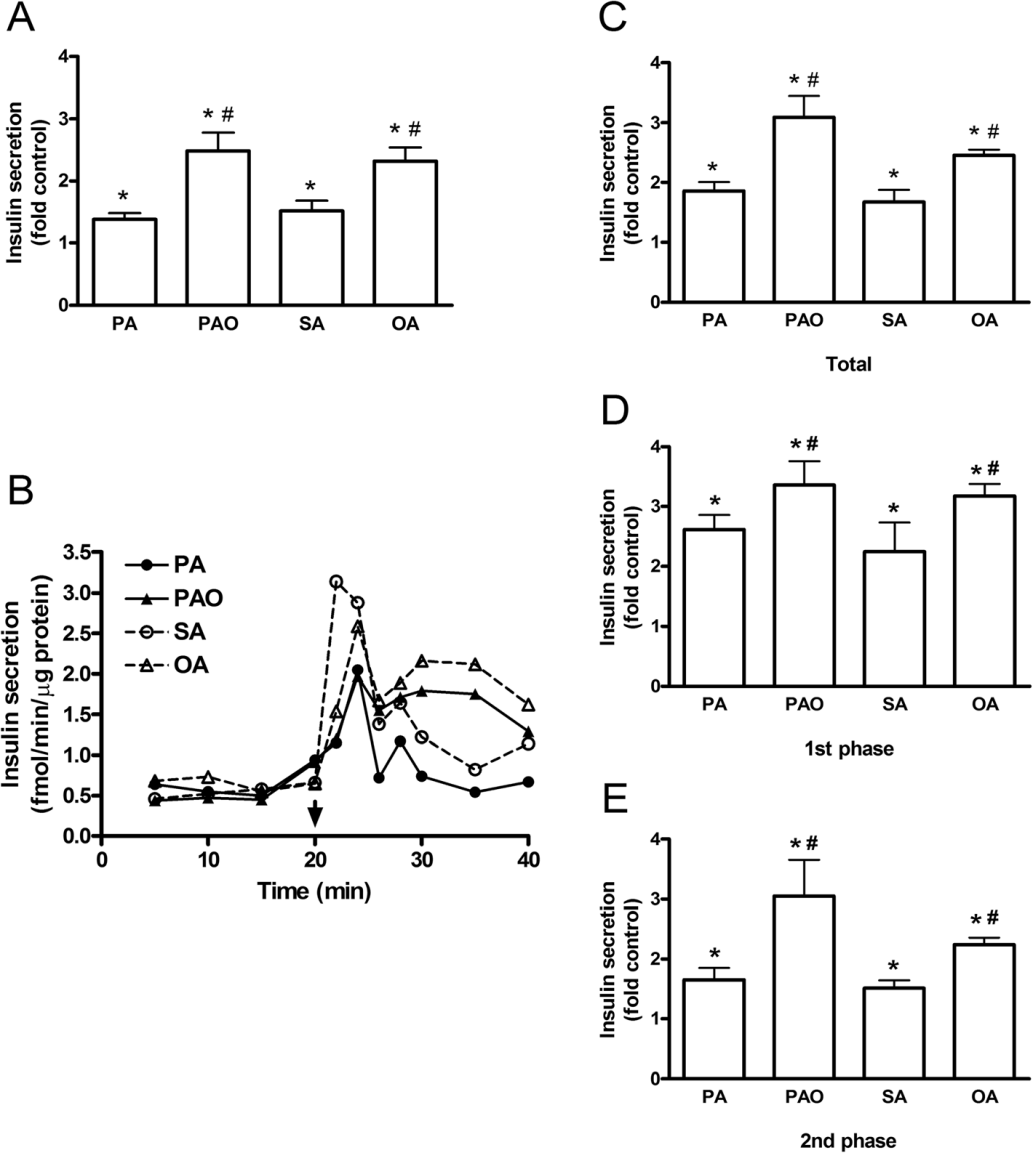
Acute effects of free fatty acids on insulin secretion in the absence of glucose. Human islets were incubated with or without palmitate (PA), palmitoleate (PAO), stearate (SA), or oleate (OA) for 20 min in the absence of glucose. **a** Insulin secretion from statically incubated human islets was expressed as fold secretion at control. **b** Representative graph of dynamic insulin secretion from perifused islets is shown. Fatty acids were added as indicated. **c**-**e** Total, first-phase, and second-phase of fatty acid-stimulated insulin secretion during perifusion was expressed as fold control. Results are means ± SEM of 8 donors. *P < 0.05 vs control, #*P* < 0.05 PA vs PAO or SA vs OA

Insulin secretion rate from statically incubated human islets in the absence of glucose was 0.59 ± 0.14 fmol/min/μg protein, which was 40 % of the secretion rate at 5.5 mmol/l glucose. When SFAs or MUFAs were included, insulin release was significantly increased with the MUFAs being more potent than the SFAs (Fig. 4a). The fold changes were approximately the same as for islets incubated in the presence of 5.5 mmol/l glucose. Dynamic insulin release was also measured by perifusing human islets in the absence of glucose. Perifused human islets secreted insulin at 0.99 ± 0.10 fmol/min/μg protein in the absence of glucose, which was 50 % of that in presence of 5.5 mmol/l glucose. Similar to the static incubation insulin release was enhanced by both the SFAs and the MUFAs with the MUFAs being more potent than the SFAs (Fig. 4b and c). Again, fold changes were similar to those observed from islets perifused in the presence of 5.5 mmol/l glucose. The increase in insulin secretion was due to rise in both first and second phases of insulin secretion (Fig. 4d and e). The results show that FFAs are capable of enhancing insulin secretion via mitochondria-independent mechanisms.

### Intracellular metabolism of fatty acids and GPR40/FFAR1 signaling are involved in the stimulation of insulin secretion by fatty acids in the absence of glucose

**Fig. 5:**
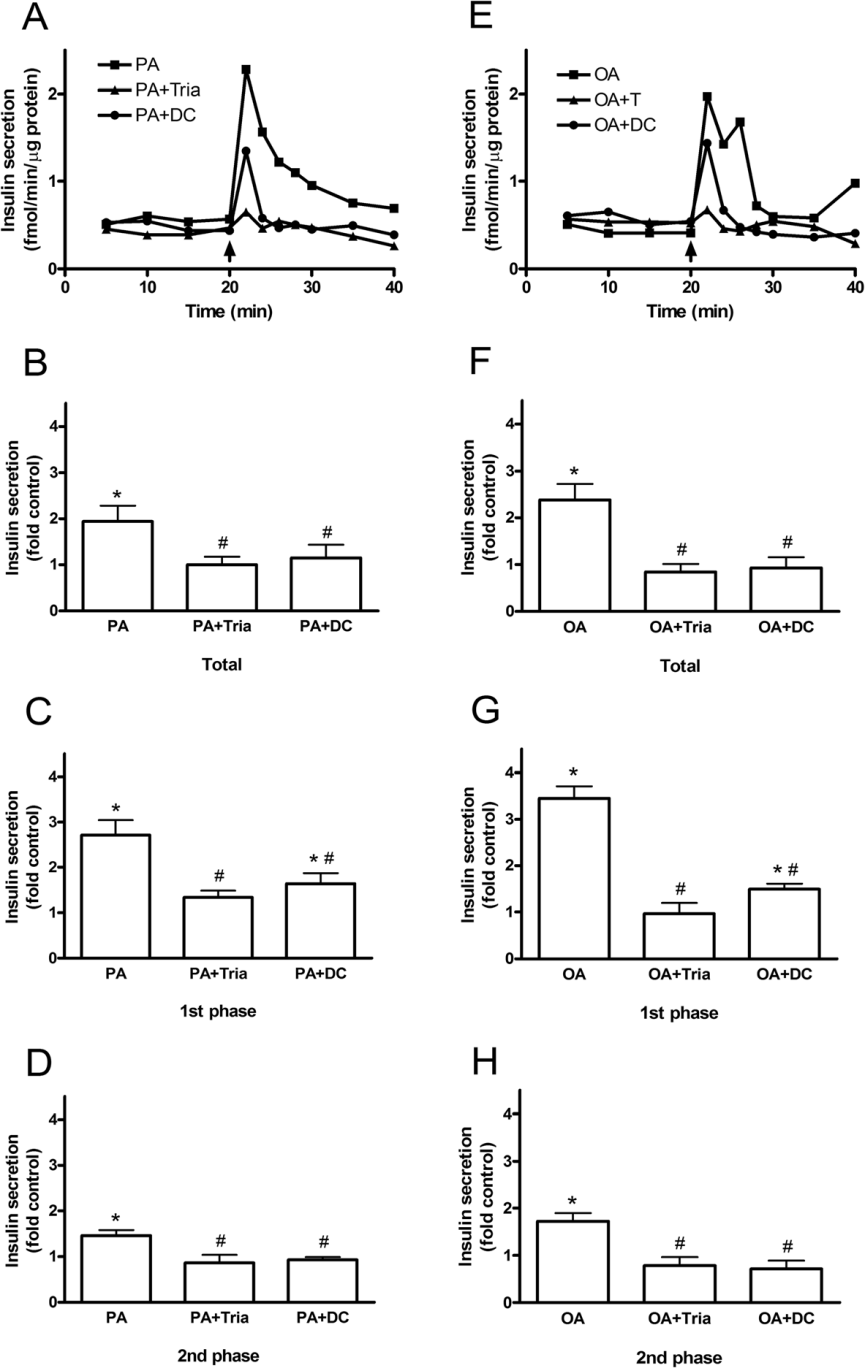
Contribution of the fatty acid metabolism and FFAR1/GPR40 signaling to FFA-induced insulin secretion in the absence of glucose. Human islets were incubated with or without palmitate (PA) (**a**-**d**) or oleate (OA) (**e**-**h**) for 20 min in the absence of glucose. **a** and **e** Representative graphs of dynamic insulin secretion from perifused islets is shown. Fatty acids were added as indicated. **b**, **c**, **d**, **f**, **g** and **h**) Total, first-phase and second-phase of FFA-stimulated insulin secretion during perifusion was expressed as fold control. Results are means ± SEM of 3 donors. *P < 0.05 vs control, #*P* < 0.05 vs fatty acid alone

To examine what are the mechanisms of mitochondria-independent action of fatty acids on insulin secretion, we inhibited intracellular metabolism of fatty acids and FFAR1/GPR40 signaling with long-chain fatty acyl CoA synthetase inhibitor triascin C and FFAR1/GPR40 inhibitor DC260126, respectively. Both inhibitors prevented palmitate-induced elevation of insulin secretion (Fig. 5a and b). The inhibitory effects of triacsin C and DC260126 on insulin secretion were also detected in oleate-treated human islets (Fig. 5e and f). The changes in insulin secretion were due to decrease in first and second phases of insulin secretion (Fig. 5c, d, g and h).

## Discussion

FFA concentrations vary widely in the circulation from hour to hour [12]. Such fluctuations take place both at high and low glucose concentrations [23]. It is generally accepted that fatty acids potentiate insulin secretion at high glucose concentrations [2, 3, 5, 16, 17]. Our study shows that fatty acids enhance insulin secretion even at low glucose concentrations. Similar to glucose, fatty acids stimulated biphasic insulin secretion [18].

In our study, MUFAs were more potent in inducing insulin secretion than SFAs, which is in contrast to previous studies [2]. In an attempt to explore the mechanisms behind the different levels of secreted insulin stimulated by different fatty acids we examined mitochondrial activity after the treatment. Mitochondrial metabolism of beta-cells is known to play a key role in maintaining nutrient-induced insulin secretion [24]. It has been demonstrated that at high glucose concentrations palmitate enhances mitochondrial function by increasing glucose oxidation [9]. In the present study we extended this observation by comparing the effects of SFAs and MUFAs on OCR in isolated human islets cultured at fasting glucose concentrations. The higher OCR observed in the presence of MUFAs was due to higher glycolysis. In line with these results, in the absence of glucose, ECAR and OCR were not elevated by the addition of fatty acids. Surprisingly, insulin secretion was induced by fatty acids even in the absence of glucose with MUFAs being stronger than SFAs. One of the potential explanations of stimulating insulin secretion but not metabolism in the absence of glucose is the action of fatty acids via FFAR1/GPR40 signaling. Indeed, inhibition of this pathway in SFA- and MUFA-treated human islets cultured in the absence of glucose reduced insulin secretion to the control level. Presumably, MUFAs have higher capacity to activate FFAR1/GPR40 signaling than SFAs. The differential potency of fatty acids to activate this receptor signaling may also explain higher OCR in the presence of MUFAs at 5.5 mM glucose. It has been demonstrated that receptor signaling affects mitochondrial respiration [9]. It needs to be mentioned that our hypothesis is not in line with a previous study where it has been reported that pEC50 values for the elevation of intracellular calcium in HEK293 cells stably expressing FFAR1/GPR40 were not significantly different between MUFAs and SFAs [25]. Reduction of insulin secretion to control level from SFA- and MUFA-treated human islets by triacsin C implies that higher capacity of MUFAs to stimulate insulin secretion might be explained even by its intracellular metabolism. One of the potential mechanisms is a greater capacity of triglyceride esterification [26, 27]. Higher esterification rate would increase generation of metabolites via the glycerolipid/free fatty acid cycle and, in such way, cause higher secretion of insulin [28]. On the other hand, in the absence of glucose, FFAs would rather be used for oxidation and production of ATP than for esterification [13]. Complete inhibition of fatty acid-induced stimulation of insulin secretion by inhibiting either intracellular metabolism or GPR40/FFAR1 signaling suggests that these pathways are interrelated and act synergistically. Interestingly, whereas OCR was reduced by approximately 10 % in islets incubated in the absence of glucose, insulin secretion was lowered by almost 60 %. It seems that beta-cells may efficiently use different sources for ATP generation and, thereby, maintaining normal cell function. However, to efficiently enhance insulin secretion glucose is required.

## Conclusions

In conclusion, we found that short-term exposure of human islets to long-chain FFAs induced insulin secretion at physiologically fasting blood glucose levels, with MUFAs being more powerful than SFAs. These effects were partly due to increased glycolytic flux and mitochondrial respiration and partly due to mitochondria-independent effects via fatty acid metabolism and FFAR1/GPR40 signaling. Our results imply that stimulatory effects of fatty acids on insulin secretion at fasting glucose concentrations may contribute to hyperinsulinemia in subjects with elevated levels of FFAs.

## Abbreviations

ECAR: Extracellular acidification rate
FFA: Free fatty acid
MUFA: Monounsaturated free fatty acid
OA: Oleate
OCR: Oxygen consumption rate
PA: Palmitate
PAO: Palmitoleate
PBS: Phosphate buffer saline
SA: Stearate
SFA: Saturated free fatty acid
